# Prevalence and incidence rate of tuberculosis among HIV-infected patients enrolled in HIV care, treatment, and support program in mainland Tanzania

**DOI:** 10.1186/s41182-020-00264-1

**Published:** 2020-09-04

**Authors:** M. Majigo, G. Somi, A. Joachim, J. Manyahi, J. Nondi, V. Sambu, A. Rwebembera, N. Makyao, A. Ramadhani, W. Maokola, J. Todd, M. I. Matee

**Affiliations:** 1 Department of Microbiology and Immunology, School of Medicine, Muhimbili University of Health and Allied Sciences, Dar es Salaam, Tanzania; 2 National AIDS Control Programme, Ministry of Health, Community Development, Gender, Elderly and Children, Dar es Salaam, Tanzania; 3 London School of Hygiene and Tropical Medicine and National Institute for Medical Research, Mwanza, Tanzania

**Keywords:** Tuberculosis, HIV, Incidence of tuberculosis, Antiretroviral therapy, Collaborative TB/HIV

## Abstract

**Background:**

Despite improvements in access to antiretroviral therapy (ART), mortality in people living with human immunodeficiency virus (PLHIV) is still high and primarily attributed to tuberculosis (TB) infection. In Sub-Saharan Africa, approximately 80% of HIV-related mortality cases are associated with TB. Relatively little is known about the incidence of TB among PLHIV in Tanzania and the determinant factors. We report the prevalence and incidence rate of confirmed TB and determine association with selected demographic and program-related factors based on data in the national HIV care and treatment program from 2011 to 2014.

**Methods:**

We used the Tanzania National AIDS Control Programme database to obtain information on all HIV clients enrolled in the HIV care and treatment program between January 2011 and December 2014. We analyzed retrospective cohort data to assess the prevalence and TB incidence rate per 1000 person-years. A multivariable Cox proportional hazards regression model was used to estimate hazard ratios and 95% confidence intervals for putatively associated factors.

**Results:**

Over 4 years, there were 22,071 confirmed cases of pulmonary TB in 1,323,600 person-years. The overall TB incidence was around 16.7 (95% CI 16.4–16.9) cases per 1000 person-years. The annual incidence rate decreased by 12.4 % from 17.0 (95% CI 16.5–17.4) in 2011 to 14.9 (95% CI 14.5–15.4) in 2014. The TB incidence rate was higher in persons not using ART and in males than in females. The incidence of TB was higher in patients with advanced HIV disease and decreased with increasing age. The overall prevalence of TB was 2.2%, with a peak prevalence of 2.5% in 2013 and was higher among children < 15 years (3.2%) in the same year.

**Conclusion:**

The study found an overall decrease in the incidence of TB in PLHIV. Our results emphasize the need for early initiation of ART and the provision of TB preventive therapy for those PLHIV without active TB after intensified TB case-finding.

## Introduction

Tuberculosis (TB) and human immunodeficiency virus (HIV) co-infection is a significant public health problem worldwide [1–3]. The lifetime risk of developing active TB among people living with HIV (PLHIV) maybe 20 times higher than in people without HIV [4]. Tanzania is among the 30 countries with high TB burden and TB and HIV co-infection in the world [5]. The National Tuberculosis and Leprosy Programme annual report of 2017 indicates that about 31% of all TB-notified cases had co-infection with HIV [6]. Despite the increased access to antiretroviral therapy (ART), mortality in PLHIV is still high, and TB is the leading cause of mortality [7–9]. Some studies found that TB preventive therapy by isoniazid reduces the incidence of TB in HIV-infected patients [10, 11]. Conversely, some studies have reported an unacceptably high incidence of TB after ART initiation as a result of immune reconstitution [12, 13].

In 2004, the World Health Organization (WHO) formulated an interim policy to guide member states in implementing collaborative TB/HIV activities [14] and further addressed the requirement for collaborative effort to address the burden of TB and HIV [15]. Subsequently, the Ministry of Health in Tanzania formed a national TB/HIV coordinating body in 2005 for delivering integrated TB and HIV services to reduce the burden of TB in PLHIV [14]. In these collaborative activities, screening for HIV among TB patients occurs in the TB clinics, likewise, the screening for TB at HIV care and treatment clinics. The country recommends TB preventive therapy to be part of the package of care for PLHIV. All HIV-positive individuals who screen negative for active TB are eligible for isoniazid preventive therapy for 6 months [16]

Previous reports from analysis of data collected between 2004 and 2012 in Dar es Salaam, the largest city in Tanzania, reported the TB incidence rate of 7.9/100 person-years before ART initiation and 4.4/100 person-years for patients receiving ART [17]. However, the reported TB incidence rates among HIV-infected patients before and after ART initiation were from HIV-infected cohort in the largest city in the country, which might not reflect the country-wide incidence rates. Also, the incidence rate might change with time, taking into consideration the implementation of collaborative TB and HIV activities. We, therefore, analyzed data in the national HIV care and treatment program collected from 2011 to 2014 to determine the country-wide incidence of TB and among PLHIV related factors.

## Material and methods

### Design, setting, and population

The descriptive-analytical study of retrospective cohort data is routinely collected between 2011 and 2014 at HIV service delivery clinics and archived in the National AIDS Control Programme database. The data included information from all clients attending HIV care and treatment services, as stipulated in the national program for HIV management [16]. All HIV patients in Tanzania receive the same quality of care and treatment services at care and treatment clinics (CTC). The clinics have been set up, in both public and private hospitals, health centers and dispensaries, throughout Tanzania. An integral part of the delivery of care and treatment is to ensure that effective monitoring and evaluation of the HIV program takes place.

### Care and treatment service

Provision of quality HIV care and treatment service was guided by the national HIV/AIDS care and treatment guideline [16]. Patients eligible for ART had baseline CD4 counts below 200 cells/mm^3^ regardless of WHO stage or baseline CD4 counts between 200 and 350 cells/mm^3^ and be in baseline WHO stage 3 or 4 or baseline WHO stage 4 regardless CD4 counts. Also, all adult patients with confirmed TB status during clinic enrolment were eligible for ART. Before the initiation of ART in any patient, health care workers conduct a complete assessment of the patient, starting with in-depth medical history followed by a head-to-toe physical examination, including WHO clinical staging. TB screening questionnaire was administered at baseline and during scheduled follow-up visits.

The country introduced a new guideline in 2012 to combat the poor enrolment of the pediatric population on ART that directed treatment of all HIV-positive children, under the age of 15 years, with suitable ART regimes [16]. Health care workers evaluated HIV-infected children for TB disease at the time of HIV diagnosis; at any time when they present with symptoms suggestive of TB during a scheduled follow-up visit or when they have a history of a new contact to an adult with TB. A standardized score chart, which is TB symptom-screening, was a tool used for screening of tuberculosis in children.

### Data management

Primary data generated in the CTC was captured on facility-held information collection tools designated as CTC2 card. Information from CTC2 cards at the health facility level is aggregated either manually or electronically at different levels up to the national level. The manually aggregated data is electronically captured at the national level. Moreover, patient-level data from health facilities with electronic CTC2 databases is exported to the national level. In the end, electronically available aggregated data at the national level and patient-level data create a macro database designated as CTC3.

### Definition of variables

Adult patients are referred to as those aged 15 years and older, as described in the National guideline for the Management of HIV and AIDS in Tanzania [16]. HIV clients “non-ART” are all patients who have enrolled in CTC but have not yet initiated ART. HIV clients “on ART” are all patients who have initiated ART. The date of enrolment in CTC was defined as the first date documented in the database, and enrolment year extracted from the first visit date. The date first initiated on ART was taken from the CTC record if it was recorded and otherwise defined as the first date recorded to receive ART. Patients were defined as the loss to follow-up (LTFU) if they had not attended any clinic in the 6 months preceding 31 December 2014 and were not known to have died. TB incidence was defined as a new bacteriologically confirmed case of TB by smear, Gen-Xpert, or culture between1 January 2011 and 31 December 2014.

### Data analysis

Abstracted data were analyzed using StataCorp 2015 (Stata Statistical Software: Release 14. College Station, TX: StataCorp LP). The individual-level data were used to calculate the prevalence, incidence rate, and risk factors for the occurrence of tuberculosis. The analysis included all individuals who attended CTC from 1 January 2011 to 31 December 2014. The prevalence of tuberculosis was calculated for the entire patient population and separately for each program year. Further analyses stratified the subjects by sex, age group, and ART status (ART and Non-ART).

Patient characteristics at the time of incidence of TB were described by the year using frequencies and percentages for categorical variables. Patients seen in 2011 were followed until one of the following happened: the first episode of TB, or censored due to death, lost-to-follow-up, or on 31 December 2014. We divided the interval into the years to obtain annual TB incidence. We calculated the TB incidence rate by dividing the total number of TB incidence by the 1000 person-year of observation. A multivariable Cox proportional hazards regression model was used to estimate hazard ratios adjusted for all covariates and 95% confidence intervals (CI) for factors associated with TB incidence. Kaplan-Meier estimation methods were used to calculate the probabilities of cumulative TB incidence after enrolment into the clinic, stratified by baseline characteristics of interest.

## Results

Data were extracted and analyzed from records of 527,249 individuals with a total of 11,539,844 clinical encounters enrolled in HIV care and treatment services between 2011 and 2014, with an average of 6 encounters per year (Table 1).

**Table 1:** Number of individuals and clinic encounters in HIV care, treatment, and support program in Tanzania from 2011 to 2014

Program year	Number of individuals	Total clinic encounters
2011	427,117	2,560,290
2012	449,114	2,565,557
2013	461,857	3,004,427
2014	527,249	3,409,570
**Total**		**11,539,844**

### Prevalence of tuberculosis

The number of cases and prevalence of TB among HIV clients enrolled in care and treatment clinics in the period of 2011 to 2014 are summarized in Table 2. The overall prevalence was 2.2%, with a peak of 2.5% in the year 2013. Noticeably higher TB prevalence was found among children < 15 years, which peaked at 3.2% in 2013. Overall, TB was more reported in males than in females, and the differences were consistent over the 4 years. The highest prevalence in males was 3.5% observed in 2013. Throughout the 4 years, there was a relatively lower prevalence of TB among PLHIV on ART than those not on ART.

**Table 2:** Prevalence of TB among individuals enrolled in HIV care, treatment, and support program in Tanzania from 2011 to 2014

Characteristics	2011	2012	2013	2014	Total
	***N*** (%)	***N*** (%)	***N*** (%)	***N*** (%)	***N*** (%)
**Overall**	8765 (2.1)	9798 (2.3)	11,212 (2.5)	9857 (1.9)	39,632 (2.2)
**Age group**					
< 15 years	687 (2.1)	784 (2.3)	1066 (3.2)	854 (2.4)	3391 (2.5)
≥ 15 years	8078 (2.1)	9014 (2.3)	10,146 (2.4)	9003 (1.9)	36,241 (2.2)
**Sex**					
Male	3858 (2.9)	4474 (3.2)	5066 (3.5)	4681 (2.8)	18,079 (3.1)
Female	4907 (1.8)	5324 (1.8)	6146 (2.0)	5176 (1.5)	21,553 (1.7)
**ART status**					
ART	1145 (1.5)	883 (1.4)	630 (1.2)	699 (1.2)	3357 (1.3)
Non-ART	7620 (2.3)	8915 (2.4)	10,582 (2.6)	9158 (2.0)	36,275 (2.3)

### Incidence of tuberculosis

We analyzed the incidence of TB by following up on patients who visited the HIV clinics beginning in each of the years from 2011 to 2014. The outcome of interest was confirmed TB. Over the 4 years, there were 22,071 confirmed cases of TB among clients attending CTC (Table 3). The overall incidence of TB was around 16.7 (95% CI 16.4–16.9) cases per 1000 person-years. The annual incidence rate per 1000 person-years was 17.0 (95% CI 16.5–17.4) in 2011 and 14.9 (95% CI 14.5–15.4) in 2014, indicating a 12.4 % decrease over the 4 years. The incidence rate was significantly lower among females, 13.9 (95% CI 13.7–14.2) per 1000 person-years than males 22.8 (95% CI 22.4–23.3) per 1000 person-years. The incidence rate of TB was fourfold more common in non-ART patients as compared to ART patients, being 47.2 (46.1–48.3) per 1000 person-years versus 12.8 (12.6–13.0) per 1000 person-years. The risk of developing TB was significantly lower in adults (hazard ration less 1) compared to children below 15 years and increased with stage of clinical HIV disease (hazard ration from 2 in stage 2 to 12 in stage 4) (Table 3).

**Table 3:** Incidence of tuberculosis among individuals enrolled in HIV care, treatment, and support program in Tanzania in 2011 to 2014

Variable	TB cases	1000 person-years	TB incident rate/1000 person-years (95%CI)	Hazard ratio (95% CI)
**Overall**	22,071	1323.6	16.7 (16.4–16.9)	-
**Year of diagnosis**				
2011	5366	316.2	17.0 (16.5–17.4)	1
2012	5254	317.9	16.5 (16.1–17.0)	1.07 (1.02–1.14)
2013	6356	348.5	18.2 (17.8–18.7)	1.15 (1.09–1.21)
2014	5095	342.3	14.9 (14.5–15.4)	0.84 (0.80–0.89)
**Sex**				
Male	9306	407.7	22.8 (22.4–23.3)	1
Female	12,764	916.9	13.9 (13.7–14.2)	0.65 (0.64–0.67)
**ART status**				
Non-ART	7026	148.9	47.2 (46.1–48.3)	1
ART	15,045	1152.7	12.8 (12.6–13.0)	0.47 (0.46–0.49)
**HIV clinical stage**				
1	844	180.0	4.7 (4.3–5.0)	1
2	2306	300.1	7.7 (7.4–8.0)	2.31 (2.09–2.55)
3	13,233	604.8	21.9 (21.5–22.2)	10.2 (9.31–11.09)
4	5214	216.7	24.1 (23.4–24.7)	12.3 (11.2–12.45)
**Age group (years)**				
< 15	2034	97.5	20.8 (20.0–21.8)	1
15–24	1210	70.4	17.2 (16.3–18.2)	0.66 (0.63–0.78)
25–34	5514	323.1	16.9 (16.4–17.3)	0.78 (0.75–0.81)
35–44	7554	458.9	16.5 (16.1–16.8)	0.93 (0.90–0.97)
45–54	3990	253.9	15.7 (15.3–16.2)	0.94 (0.90–0.98)
55+	1754	116.9	15.0 (14.3–15.7)	0.87 (0.83–0.92)

### Cumulative probability of TB incidence

**Fig. 1:**
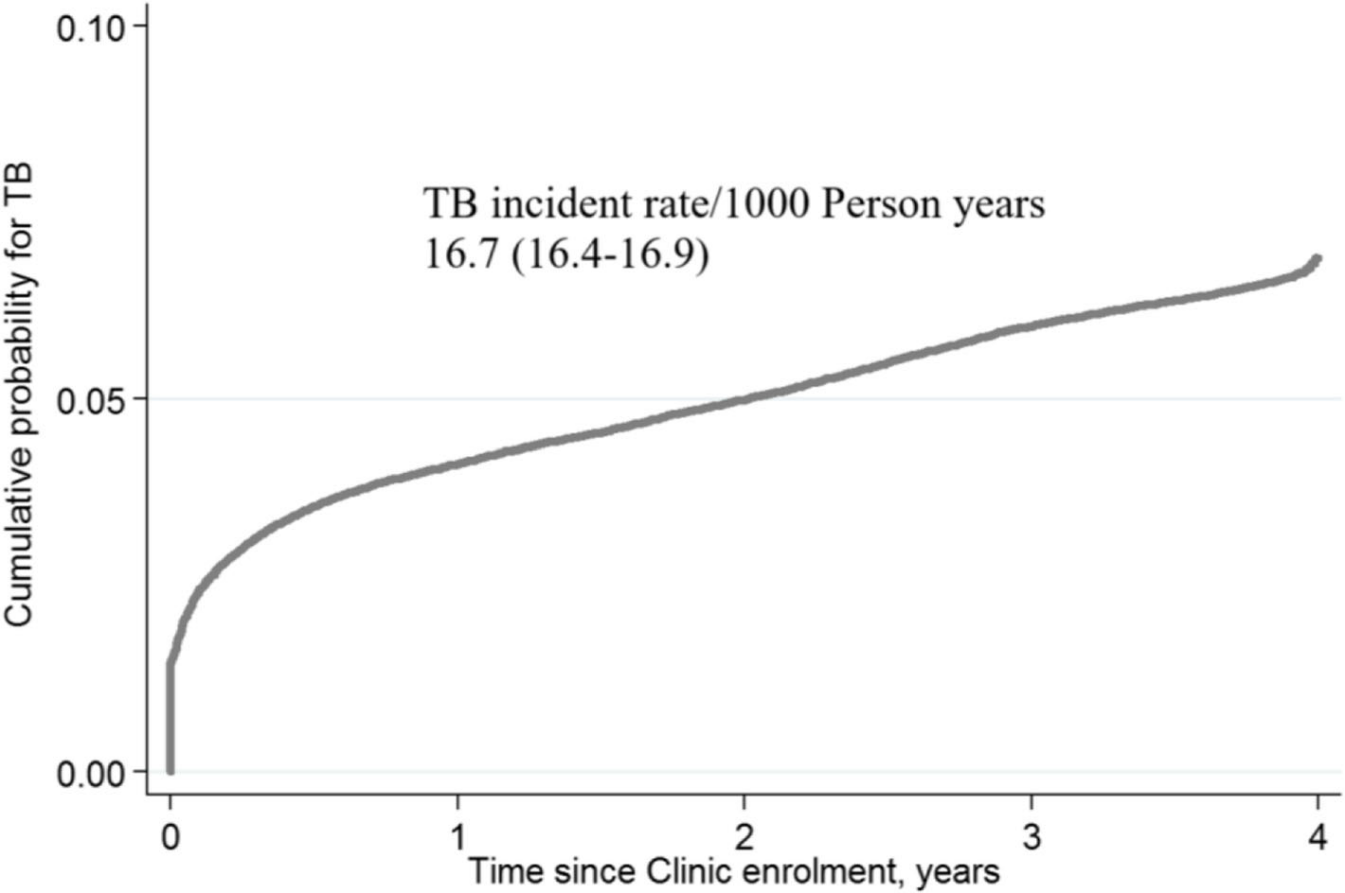
Cumulative probability of TB incident after enrollment to HIV care and treatment

**Fig. 2:**
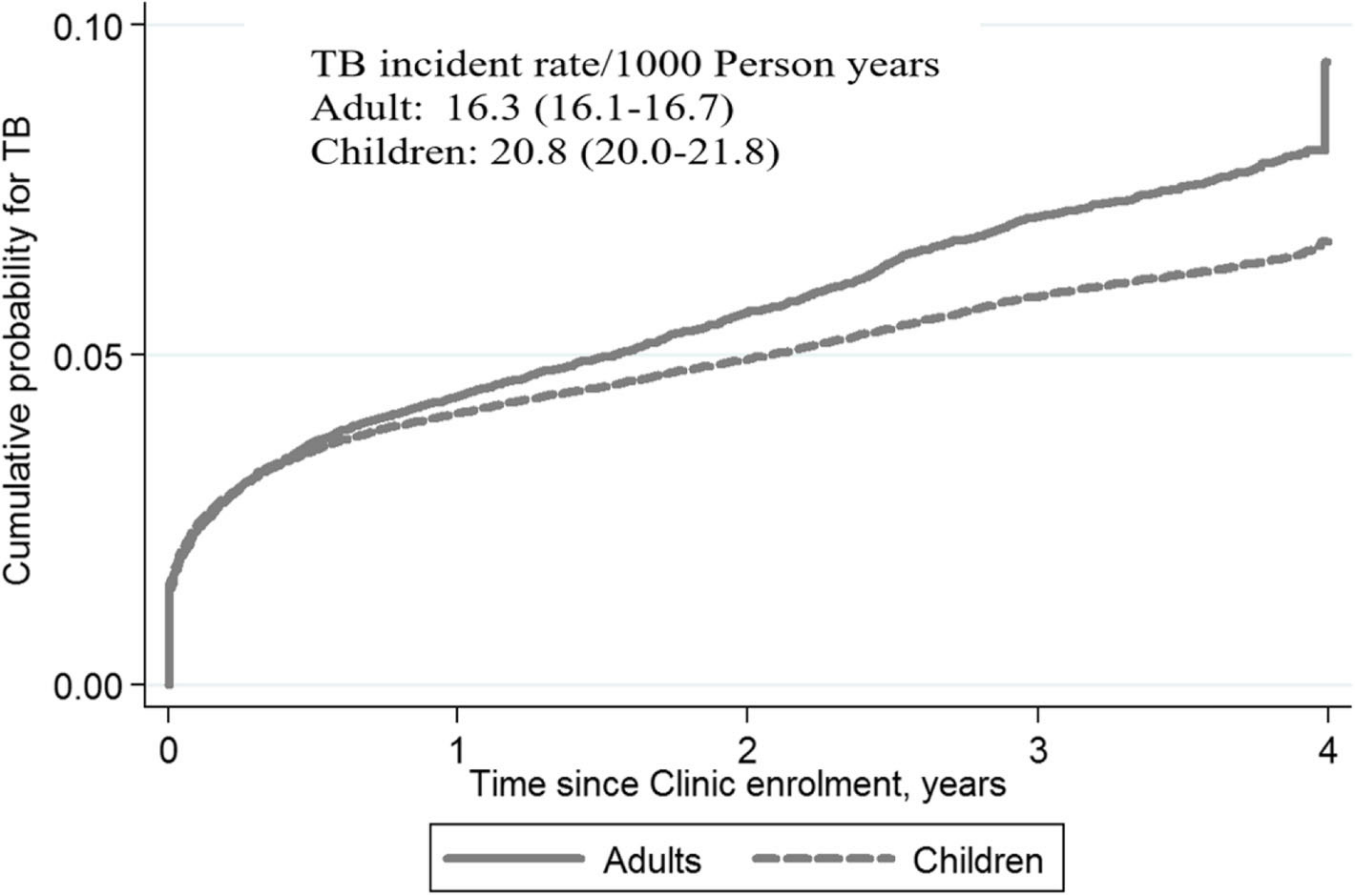
Cumulative probability of TB incident after enrollment to HIV care and treatment by age

**Fig. 3:**
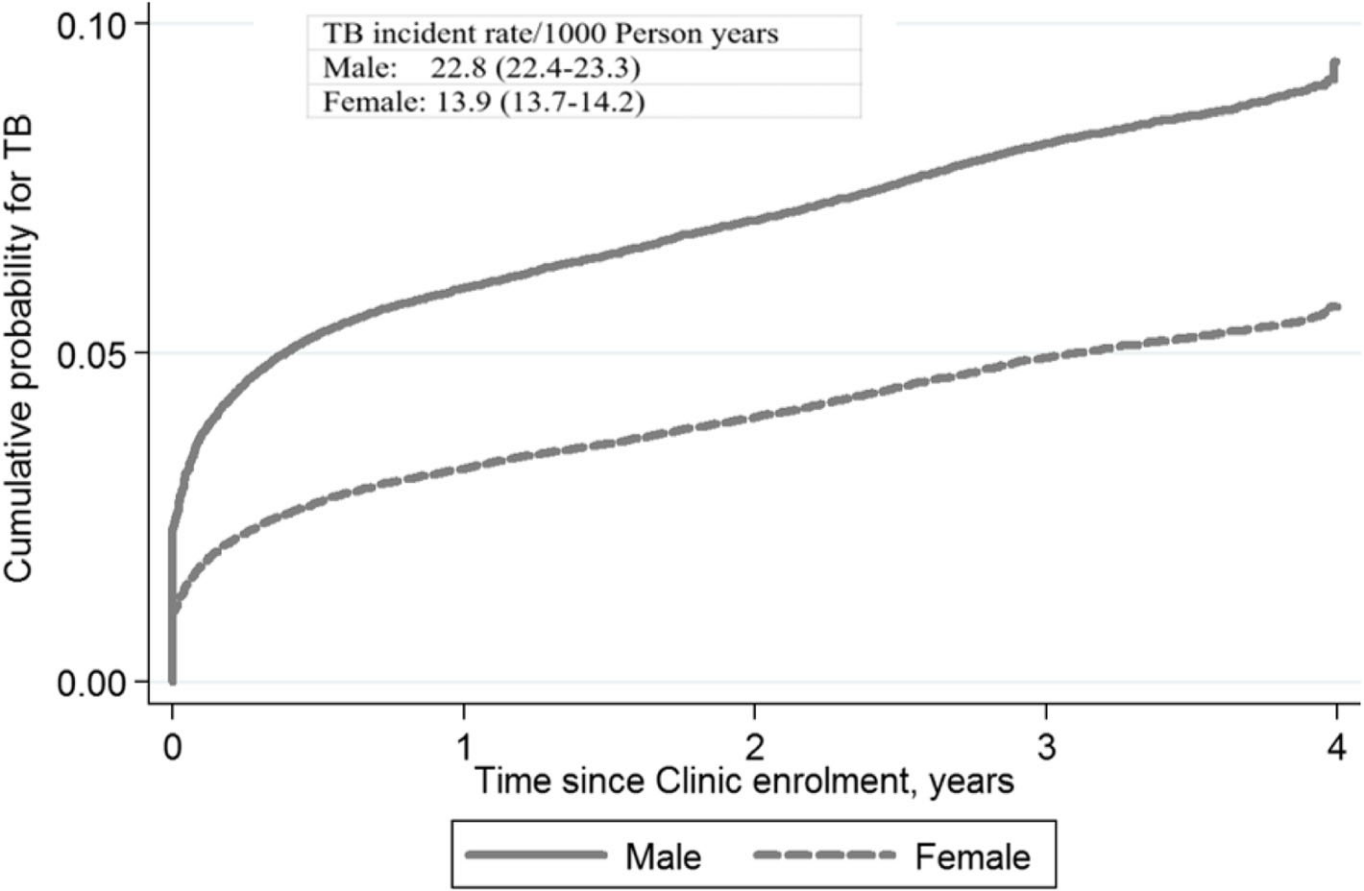
Cumulative probability of TB incident after enrollment to HIV care and treatment by age and sex

Kaplan-Meier curves were used to assess the cumulative probability of TB incidence over time and stratified by baseline variables of age and sex. The overall probability of TB incidence was 50% by 2 years since enrollment and reached around 65% in 4 years. The hazard of TB incidence was highest in the first year of enrolment (Fig. 1). The cumulative probability of TB incidences was similar between adults and children in the first 6 months, progressively increasing more in adults than children after 6 months. Two years after, the cumulative enrollment probability for TB incidence was 50% in children while it reached around 60% among adults (Fig. 2). There was a clear difference in the cumulative probability of TB incidence between males and females. By 6 months, the cumulative probability of TB incidence among males reached 50% compared to 25% among females, who reached 50% after 3 years (Fig. 3).

## Discussion

We conducted an assessment of the occurrence of a first episode of pulmonary tuberculosis (PTB), involving individuals whose first visits at HIV care and treatment services occurred between January 2011 and December 2014. As a result, the study included a total of 527,249 individuals with a total of 11,539,844 clinical encounters at health facilities implementing TB and HIV collaborative activities. The overall TB incidence rate was around 16.7 (95% CI 16.4–16.9) cases per 1000 person-years with annual incidence rate decreasing from 17.0 to 14.9 per 1000 person-years, representing around 12% decrease over the 4 years.

The observed incidence of TB in PLHIV on ART reported in this study (12.8 per 1000 person-years) is on the lower side compared to observed incidences in prior studies conducted among adults in Tanzania [17]. The finding may be a reflection of continuous improvement in HIV and TB collaborative activities and the reduction of TB incidence in HIV programs. In support, we observed in this analysis that in patients receiving ART, the decrease in TB incidence went from 13.5 (95% CI 13.1–14.0) per 1000 person-years in 2011 to 11.0 (95% CI 10.6–11.3) in 2014 a decrease of 19% over the 4 years. In contrast, those not on ART, the TB incidence went from 33.3 (95% CI 31.8–34.8) per 1000 person-years in 2011 to 104.9 (95% CI 99.7–110.3) per 1000 person-years in 2014, a three-fold increase in the incidence of TB. The reported incidences in other parts of African countries range from 0.9 to 7.9 cases per 100 PY [18–21].

As observed in this analysis and reported in other studies, the use of ART has a significant advantage on the reduction of incidence of TB, evidenced by a marked low incidence of TB among persons on ART compared to those not on ART [17, 22, 23]. The findings are conceivable because the TB infection and reactivation have been associated with the degree of immune suppression, which improves after the start of ART [24]. In individuals not on ART, HIV infection increases susceptibility to TB and is the most potent factor in transferring latent or recently acquired TB infection to active clinical diseases [25, 26]. Our findings emphasize the need for TB preventive therapy, which has been found to reduce the risk of developing tuberculosis and prolongs survival [27].

The TB incidence in the current study was significantly associated with advanced HIV disease defined by WHO clinical stages. Our findings are consistent with previous findings and support the known benefit of early HIV diagnosis and treatment [28]. There is a much higher risk of HIV and TB co-infected patients to develop active TB either from the latent infection or rapid progression of a new infection, especially in advanced HIV disease [29]. TB incidence as a result of immune reconstitution after initiation of ART is likely to occur in advanced HIV disease [12, 13]. Although this study did not record immune reconstitution inflammatory syndrome, the majority of TB incidence among patients with advanced HIV disease occurred within 2 months after ART initiation, as previously observed [12, 13]

The study found a higher risk of TB among males compared to females, which is comparable to other studies [2, 30, 31]. The most probable reason to explain this difference could be the biological differences in disease and disease presentation, which favors women [32, 33]. Furthermore, men are more likely to report predisposing factors for TB, like smoking than females [34]. PLHIV, aged below 15 years, had a significantly higher risk of TB incidence than adults. However, encouragingly, the observed overall incidence of TB in children (20.8 cases per 1000 PY) is less compared to findings from a previously reported cohort study in Tanzania (5.2 cases per 100 PY) [35]. This signifies the effort in increasing access to pediatric HIV and TB care and treatment in Tanzania [36].

The major strength of this study is that it drew on a large and nationally representative sample from a country with a significant burden of TB and HIV. It has provided some insights into the situation of TB in clinical services for HIV care and treatment in Tanzania. Nevertheless, the analysis excluded patients without bacteriological confirmation, which lead to underestimation of the magnitude of TB. Besides, many of our facilities may not be able to perform a bacteriological diagnostics test, which has high sensitivity and specificity, instead of depending on radiological and clinical diagnoses. The other limitation is that the data come from the electronic CTC database, which is used in the larger and better run clinics. The situation reported here might not be the same in smaller health facilities that have not provided electronic data.

## Conclusion

The study found an overall decrease in the incidence of TB in PLHIV and demonstrated more among those on ART, adult, female, and low HIV clinical stage. Our results reinforce the recommendation of HIV test and treat, intensified TB case findings, and TB preventive therapy for those PLHIV without active TB.

## Data Availability

The raw data supporting the findings can be accessed on reasonable request to the Head of the Epidemiology Unit of the National AIDS Control Programme.

## Abbreviations

ART: Antiretroviral therapy
CTC: Care and treatment clinics
HIV: Human immunodeficiency virus
PLHIV: People living with HIV
TB: Tuberculosis
WHO: World Health Organization
